# Application of Microarray and Functional-Based Screening Methods for the Detection of Antimicrobial Resistance Genes in the Microbiomes of Healthy Humans

**DOI:** 10.1371/journal.pone.0086428

**Published:** 2014-01-22

**Authors:** Roderick M. Card, Philip J. Warburton, Nikki MacLaren, Peter Mullany, Elaine Allan, Muna F. Anjum

**Affiliations:** 1 Department of Bacteriology, Animal Health and Veterinary Laboratories Agency, Addlestone, Surrey, United Kingdom; 2 Department of Microbial Diseases, Eastman Dental Institute, University College London, London, United Kingdom; Cairo University, Egypt

## Abstract

The aim of this study was to screen for the presence of antimicrobial resistance genes within the saliva and faecal microbiomes of healthy adult human volunteers from five European countries. Two non-culture based approaches were employed to obviate potential bias associated with difficult to culture members of the microbiota. In a gene target-based approach, a microarray was employed to screen for the presence of over 70 clinically important resistance genes in the saliva and faecal microbiomes. A total of 14 different resistance genes were detected encoding resistances to six antibiotic classes (aminoglycosides, β-lactams, macrolides, sulphonamides, tetracyclines and trimethoprim). The most commonly detected genes were *erm*(B), *bla*_TEM_, and *sul2*. In a functional-based approach, DNA prepared from pooled saliva samples was cloned into *Escherichia coli* and screened for expression of resistance to ampicillin or sulphonamide, two of the most common resistances found by array. The functional ampicillin resistance screen recovered genes encoding components of a predicted AcrRAB efflux pump. In the functional sulphonamide resistance screen, *folP* genes were recovered encoding mutant dihydropteroate synthase, the target of sulphonamide action. The genes recovered from the functional screens were from the chromosomes of commensal species that are opportunistically pathogenic and capable of exchanging DNA with related pathogenic species. Genes identified by microarray were not recovered in the activity-based screen, indicating that these two methods can be complementary in facilitating the identification of a range of resistance mechanisms present within the human microbiome. It also provides further evidence of the diverse reservoir of resistance mechanisms present in bacterial populations in the human gut and saliva. In future the methods described in this study can be used to monitor changes in the resistome in response to antibiotic therapy.

## Introduction

The human body serves as a host for a diverse range of commensal and symbiotic microorganisms, collectively termed the microbiota. The microbiota is a natural component of the human host that is acquired from birth onwards, and has important roles in nutrition, development of the immune system, and protection from colonisation by pathogens [1], [2]. The microbiota can also play a role in disease, as some members are opportunistic pathogens that are capable of inducing disease following a disturbance or disruption to their host (e.g. disease, wound or medication) [3]. The microbiota contributes a small but significant proportion to the host’s total mass and is estimated to contain ∼10 fold more cells and ∼100 fold more genes than the human host [2]. The microbiome is the aggregate collection of genes within the microbiota and the portion which encodes resistance to antibiotics has been termed the resistome [4].

Although there is evidence that antimicrobial use in humans and animals has had an impact upon the composition of the microbiome [5], antimicrobial resistance (AMR) genes have been detected in humans, animals, and in environments where there is little or no evidence of antibiotic use by man [6]–[8]. However, it is worth noting that in the latter study by Pallecchi et al [6]–[8] it was concluded that the resistances seen in these remote communities arose not due to an independent in situ selection but due to dissemination of resistant bacteria and resistant genes from antibiotic exposed settings, indicating the indirect effect of antibiotic usage and exposure. The resistome is important in that it acts as a reservoir of AMR genes that can reside in commensals or opportunistic pathogens and can be acquired by pathogens via horizontal gene transfer, and consequently has the potential to interfere with therapeutic options following infection. The isolation of resistant bacteria by culture and subsequent elucidation of resistance mechanisms has provided insight into the AMR gene carriage of indicator organisms [8]–[11], however, as the majority of the bacteria in the microbiota cannot be readily cultivated, three approaches that do not rely on the culture of isolates have been employed to study the resistome (reviewed in [12], [13]). PCR has been used to detect known AMR genes in the resistome, in a target-based approach (reviewed in [12]). In a sequenced-based approach, the microbiome is shotgun sequenced and AMR genes identified by homology to known genes in reference databases (reviewed in [13]). These two methods only enable the detection of previously characterised genes and therefore cannot fully explore the capacity of the resistome. In functional-based screening, DNA prepared from the microbiota of a particular ecological niche is ligated into a vector and transformed into a heterologous host. The resultant clones are screened for resistance to selected antibiotics. This approach enables resistance genes to be identified without prior knowledge of their sequence (reviewed in [14]) and has been used to recover known and novel AMR genes from, for example, soil [15], [16], an activated sludge microbial community [17] and the human microbiome [18], [19].

The aim of this study was to screen the saliva and faecal resistomes of healthy adult human volunteers for the presence of AMR genes using target- and functional-based approaches. The target-based approach employed a microarray capable of detecting over 70 AMR genes in a single operation [20], to screen the resistomes for a wide range of known, clinically important resistance genes. This microarray has been used previously to study bacterial isolates in epidemiological studies [21], [22]. For the functional-based approach, clones were screened for expressed resistance to ampicillin and sulphonamide. In order to place detected AMR genes within the context of the microbiota, the microbial profiles of the samples studied were determined using 454 pyrosequencing of 16S rRNA gene amplicons.

## Methods

### Samples

The saliva and faecal samples employed in this study were collected from five European countries (Finland, France, Italy, Norway, and Scotland) as part of the EU FP6 Quality of Life Management of Living resources QLK2-CT2002-00843 “Antimicrobial resistance transfer from and between Gram-positive bacteria of the digestive tract and consequences for virulence” project and have been described previously [23]. In brief, samples of faeces and saliva were pooled from 20 healthy adult volunteers in each country, who had not received antibiotic therapy in the previous three months. DNA was prepared from the samples using the Puregene DNA extraction kit (Gentra Systems supplied by Flowgen, Nottingham, UK) as described previously [23]. Volunteers were given information on the study and all gave informed consent [23], approval for the study in Scotland was provided by the Grampian Research ethics committee (approval number LREC NoL 003//060).

### Microarray Procedure and Validation PCR

For each DNA preparation (see Table S1 for DNA concentrations), 2.5 µl was amplified using the Illustra GenomiPhi HY DNA Amplification Kit (GE Healthcare Life Sciences, Little Chalfont, UK) according to the kit protocol. The amplified DNA (6.5 µl) was then labelled in a linear multiplex reaction and added to the microarrays for hybridisation, with signals from the hybridisation duplex read on an ArrayMate (Alere Technologies, Jena, Germany) using IconoClust software (Standard version; Alere Technologies), as already described [20]. Mean signal intensities of two replicate spots per probe were used for analysis. Intensities of ≥0.2 were considered positive. The sensitivity of the amplification and microarray method employed was estimated using a dilution series of DNA extracts (25 ng to 0.025 ng) from two *E. coli* strains of known AMR gene content (strains E111592 and 01-2571) in 500 ng calf thymus DNA (Sigma-Aldrich, Dorset, UK), and the presence/absence of the expected genes at each dilution was determined. PCR was performed on amplified DNA samples for four genes using previously published primers to validate the array approach: *bla*_IMP_, *bla*_TEM_, *erm*(B), and *sul2* [20], [22].

### Library Construction and Functional-based Screening

A bacterial artificial chromosome (BAC) library was constructed as described previously [24]. Briefly, the DNA prepared from saliva samples from Finland, Italy, Norway, and Scotland was pooled and partially digested with *Hin*dIII before ligation into pCC1BAC using the CopyControl Ligation kit (Epicentre Biotechnologies, Madison, WI, USA) according to the product protocol. Ligations were transformed into electrocompetent *E. coli* TransforMax EPI300-T1^R^ cells (Epicentre Biotechnologies), according to the product protocol and, following addition of SOC medium, were allowed to recover for 1 hour at 37°C with shaking horizontally at 225 rpm. Functional-based screening was performed by plating the transformation reactions on Luria Bertani (LB) agar with chloramphenicol (12.5 µg/ml) and either ampicillin (25 µg/ml) or sulfamethoxazole (250 µg/ml) as appropriate and subsequent incubation at 37°C. Plates were checked at 24 and 48 hours after plating and resistant clones were recovered and propagated at 37°C under the appropriate selection (ampicillin at 25 µg/ml or sulfadiazine at 1024 µg/ml). The transformation reaction was also plated on LB agar with chloramphenicol (12.5 µg/ml), IPTG (0.1 M) and Xgal (40 µg/ml) as controls. Based on these controls and the estimated average insert size of 20 kb (unpublished data), the amount of DNA surveyed in the ampicillin and sulphonamide functional-based screens was estimated as 214 Mbp and 148 Mbp, respectively.

### Susceptibility Testing of Recovered Clones

Recovered clones were tested for their susceptibility to a panel of 12 antimicrobials (amikacin, amoxicillin/clavulanic acid, ampicillin, cefotaxime, ceftazidime, chloramphenicol, ciprofloxacin, gentamicin, nalidixic acid, streptomycin, trimethoprim/sulphamethoxazole 1∶19, and sulphonamide compounds) using the British Society for Antimicrobial Chemotherapy (BSAC) disc diffusion technique [25]. Susceptibility was defined using the BSAC clinical breakpoints (the legacy breakpoint was used for streptomycin), except with the sulphonamide compounds disc for which the historical AHVLA veterinary breakpoint was used [26]. Antimicrobial susceptibilities of the reference strains *E. coli* EPI300 and *E. coli* EPI300 carrying an empty pCC1BAC vector were also determined. *E. coli* EPI300 is inherently resistant to streptomycin (conferred by a mutation in the *rpsL* gene) and trimethoprim (engineered in as part of the *trfA* integration) (personal communication F. Hyde, Epicentre Biotechnologies). The pCC1BAC vector has a chloramphenicol selectable marker.

### BAC DNA Preparation, Sequencing and Analysis

Clones were cultured in LB medium supplemented with chloramphenicol (12 µg/ml) and either ampicillin (25 µg/ml) or sulfadiazine (1024 µg/ml) as appropriate. For BAC DNA preparation, 1 ml of an overnight culture was added to 9 ml LB medium with antibiotics and 10 µl copy control induction solution (Epicentre Biotechnologies), then incubated at 37°C for 4 hours according to the manufacturer’s protocol. BAC DNA was recovered using the Qiaprep Spin Miniprep kit (Qiagen, Crawley, UK) according to the kit protocol for low copy number plasmids. The purified BAC DNA was fragmented by nebulization and purified using Qiaquick purification columns (Qiagen, Crawley, UK). Ends were repaired and 454-specific sequencing adapters ligated using a Rapid Library Kit (Roche Diagnostics Ltd, Burgess Hill, UK). The resultant library was sequenced on a Roche 454 GS FLX according to the manufacturer’s instructions (Roche Diagnostics Ltd). The sequence reads were filtered for quality and contigs generated using GSAssembler (v2.6, Roche Diagnostics Ltd), using the manufacturer’s default settings. The cloned DNA was trimmed of pCC1BAC host sequence. The RAST server [27] was used to identify and annotate putative open reading frames (ORFs) present in the insert DNA. The taxonomical classification of each cloned DNA was determined by sequence homology and ORF synteny using the RAST sequence-based comparison tools [27]. The Basic Local Alignment Search Tool (BLAST) was additionally used to annotate ORFs [28]. Predicted amino acid sequences of ORFs were aligned using the ClustalV method of MegAlign (Lasergene software, DNASTAR, Madison, WI, USA).

### Determining the Composition of the Microbiotas

The taxonomic diversity present in the samples was assessed by high-throughput sequencing of partial 16S rDNA gene amplicons on a Roche 454 GS FLX platform. For this, the DNA extracted from each sample was quantified and amplified with barcoded universal primers for the V4 and V5 regions of the 16S rRNA gene as described previously [29]. The Qiime pipeline version 1.5.0 [30] was used to process and analyse the 16S rRNA sequence data. Sequences were binned by samples using the sample-specific barcode sequences, trimmed of the barcode and primer sequences, filtered (sequences required a length ≥300 bp, no undetermined bases, and a perfect match to the barcode and PCR primer), and denoised. Sequences were clustered into operational taxanomic units (OTUs) using UCLUST [31] with a 97% sequence identity threshold. Chimeric sequences were identified with ChimeraSlayer [32] and excluded from further analysis. OTUs were assigned taxonomy using the Ribosomal Database Project (RDP) classifier (minimum confidence of 80%) [33] and the Greengenes database [34]. Based on the number of sequences obtained per sample (see results), the relative OTU abundance for each sample was determined at an even depth of 11070 sequences per sample (randomly picked without replacement; OTUs observed less than five times were excluded from this analysis).

## Results

### DNA-DNA Hybridisation-based Screen: Microarray of Microbiomes

The sensitivity of the microarray method used was estimated using spiked samples, and for two of the three replicates, the majority (≥70%) of the expected genes were detected when the spike was present at 0.25 ng (Table S2). Although probes had differing sensitivities, and some were positive only at higher concentrations, no false positive results were obtained. This indicates that, using this system, a bacterial AMR gene is detectable if it comprises 0.05% of the total DNA in the test sample. The saliva and faecal human DNA samples were tested using this approach and AMR genes were detected in all samples (Table 1; see Table S3 for all microarray results). Across all samples, 14 different AMR genes were detected encoding resistances to six antibiotic classes (aminoglycosides, β-lactams, macrolides, sulphonamides, tetracyclines and trimethoprim; Table 1). The average number of genes detected per sample was four (range 1–8), encoding resistances to an average of three antibiotic classes (range 1–6). The most commonly detected gene was *erm*(B), encoding macrolide resistance, which was detected by microarray in all ten samples and confirmed by PCR in eight samples (two were not tested), as previously reported [23]. The macrolide resistance genes, *vatE* and *ereA,* were each detected in a single sample only. The sulphonamide resistance gene, *sul2,* was the second most common gene and was detected in both the saliva and faecal samples from France, Italy and Norway. PCR verified the presence of *sul2* in all these microarray positive samples and in three microarray negative samples (Finland saliva, Finland faeces, and Scotland faeces). The β-lactamase gene, *bla*_TEM_, was detected by microarray in five samples. PCR verified the presence of *bla*_TEM_ in these samples and additionally detected *bla*_TEM_ in four samples (Scotland saliva, Italy faecal, Norway faecal, and Scotland faecal). Sequence analysis of six of the *bla*_TEM_ amplicons showed that they were not Extended Spectrum β-lactamase variants (three not sequenced; data not shown). The only other β-lactamase detected was *bla*_CMY/MOX_ in one sample. The β-lactamase *bla*_IMP_ is represented on the microarray by six probes and at least four are required to be positive for the gene to be considered present. In four samples, only one *bla*_IMP_ probe had a signal >0.2 and therefore this gene was recorded as absent (PCR verified that these samples were negative for *bla*_IMP_). Tetracycline resistance genes were detected in six of the ten samples tested, *tet*(B) was detected only in saliva samples and *tet*(X) was detected mainly in faecal samples. Five different aminoglycoside resistance genes were detected: *strA* and *strB* in faecal samples; *aadB*, *aac6′-aph2′*, and *aac6′-Ib* in saliva samples. Additionally, one trimethoprim resistance gene (*dfrA14*) was detected by microarray.

**Table 1:** Antimicrobial resistance genes detected by microarray in human saliva and faecal microbiomes.

Antibiotic Class	Finland Saliva	France Saliva	Italy Saliva	Norway Saliva	Scotland Saliva	Finland Faecal	France Faecal	Italy Faecal	Norway Faecal	Scotland Faecal
Aminoglycoside	*aadB*	*aac6′-aph2*′; *aadB*	not detected	not detected	*aac6′-aph2′*; *aac6′-Ib*	not detected	*strB*	*strA*; *strB*	not detected	not detected
Beta-lactam	*bla*_TEM_	*bla*_TEM_	*bla*_TEM_	*bla*_TEM_	not detected^1^	*not detected*	*bla*_CMY/MOX_; *bla*_TEM_	not detected^1^	not detected^1^	not detected^1^
Macrolide	*erm(B)*	*erm(B); vatE*	*erm(B)*	*erm(B)*	*erm(B)*	*erm(B)*	*ereA; erm(B)*	*erm(B)*	*erm(B)*	*erm(B)*
Sulphonamide	not detected^2^	*sul2*	*sul2*	*sul2*	not detected	not detected^2^	*sul2*	*sul2*	*sul2*	not detected^2^
Tetracycline	*tet*(B)	*tet*(B); *tet*(X)	not detected	*tet*(B)	not detected	not detected	*tet*(X)	*tet*(X)	not detected	*tet*(X)
Trimethoprim	not detected	not detected	not detected	not detected	not detected	not detected	*dfrA14*	not detected	not detected	not detected
Total number of AMR genes detected	4	8	3	4	3	1	8	5	2	2
Number of antibiotic classes	4	5	3	4	2	1	6	4	2	2

1 PCR positive for *bla_TEM_*.

2 PCR positive for *sul2*.

### Functional-based Screen: Ampicillin

Five clones were recovered and propagated from the ampicillin functional-based screening. The antimicrobial susceptibilities of each clone were tested by disc diffusion, and three had intermediate resistance to ampicillin (Table 2). The BACs from these three clones were purified and sequenced. The size of the inserts ranged from 9,476 bp to 16,716 bp and contained 7 to 13 predicted ORFs (Table 2). The cloned DNA in each BAC had high homology (93–96% nucleotide sequence identity) and gene synteny to the *Haemophilus parainfluenzae* genome. The clones spanned, to differing extents, the same region of the *H. parainfluenzae* genome. Six ORFs were shared by all three clones and within this region, three ORFs with sequence homology to the *acrRAB* operon were identified. The genes *acrA* and *acrB* encode components of a multidrug efflux pump with a broad substrate range, including ampicillin [35], and *acrR* encodes a transcriptional repressor of the *acrRAB* operon [36], [37]. The identity between the predicted amino acid sequences of the cloned *acrA* and *acrB* genes and that in *H. parainfluenzae* was ≥98.7% and ≥98.4% respectively, while for *acrR* the identity was ≥88.5%, and there were no mutations causing frame shifts or early translation termination. The three remaining ORFs shared by the clones do not have predicted functions related to ampicillin resistance and putatively encode a primosomal protein N’ (*PriA*), a cell division protein (*FtsN*), and a membrane-bound protease (*HtpX*). Consequently, the *acrRAB* operon is predicted to confer the reduced susceptibility to ampicillin observed in the three clones. Clone AMP7 contained an IS*5* element, which was not present in the other two AMP clones and which was 100% identical in nucleotide sequence to IS*5* elements from *E. coli* and is assumed to have transposed into the insert from the genome of the *E. coli* host.

**Table 2:** Summary of BAC clones made from human Saliva DNA recovered from functional-based screens.

Clone ID^1^	Antibiotic employedin screen	Best match taxonomic classification ofcloned DNA (Accession number)	NucleotideIdentity (%)	Size of ClonedDNA (bp)	Predictednumber of ORFsin cloned DNA^2^	Antibiotic Susceptibilities^3^	Gene(s) responsible for resistance phenotype
AMP4	Ampicillin	*Haemophilus parainfluenzae* (NC_015964)	96	9,476	7	Amp^I^	*acrRAB*
AMP5	Ampicillin	*Haemophilus parainfluenzae* (NC_015964)	95	12,200	10	Amp^I^	*acrRAB*
AMP7	Ampicillin	*Haemophilus parainfluenzae* (NC_015964)	93	16,716	13	Amp^I^	*acrRAB*
SUL6	Sulphonamide	*Neisseria subflava* (ACEO02000001)	96	13,526	16	Sul^RS^/Sxt^R^	*folP*
SUL8	Sulphonamide	*Neisseria subflava* (ACEO02000001)	95	14,125	18	Sul^RS^/Sxt^R^	*folP*
SUL9	Sulphonamide	*Neisseria subflava* (ACEO02000001)	96	10,250	11	Sul^RS^/Sxt^R^	*folP*
SUL15	Sulphonamide	*Neisseria subflava* (ACEO02000001)	96	11,916	13	Sul^RS^/Sxt^R^	*folP*
SUL11	Sulphonamide	*Streptococcus infantis* (NZ_AEDY01000064)	94	13,436	16	Sul^RS^/Sxt^R^	*folP*
SUL3	Sulphonamide	*Veillonella parvula* (CP001820)	88	17,734	18	Sul^R^/Sxt^RS^	*folP*
SUL5	Sulphonamide	*Veillonella parvula* (CP001820)	87	21,161	20	Sul^R^/Sxt^RS^	*folP*
SUL10	Sulphonamide	*Veillonella parvula* (CP001820)	86	15,616	16	Sul^RS^/Sxt^R^	*folP*
SUL20	Sulphonamide	*Veillonella parvula* (CP001820)	86	15,605	16	Sul^RS^/Sxt^R^	*folP*

1 Accession numbers for the clone sequences are: AMP4 (KF982313), AMP5 (KF982314), AMP7 (KF982315), SUL3 (KF982316), SUL5 (KF982317), SUL6 (KF982318), SUL8 (KF982319), SUL9 (KF982320), SUL10 (KF982321), SUL11 (KF982322), SUL15 (KF982323), and SUL20 (KF982324).

2 ORF prediction by RAST server [27].

3 Amp^I^ = intermediate ampicillin resistance; Sul^R^ = resistant to sulphonamide compounds; Sul^RS^ = reduced susceptibility to sulphonamide compounds compared to EPI300; Sxt^R^ = resistant to trimethoprim/sulphamethoxazole 1∶19; Sxt^RS^ = reduced susceptibility to trimethoprim/sulphamethoxazole 1∶19 compounds compared to *E. coli* EPI300.

### Functional-based Screen: Sulphonamide

From the sulphonamide functional-based screen a total of 23 resistant clones were recovered. The antimicrobial susceptibilities of these clones were determined by disc diffusion. Seven clones (SUL6, 8, 9, 10, 11, 15, and 20) were resistant to trimethoprim/sulphonamide, and had reduced susceptibility (but not clinical resistance) to sulphonamide compounds when compared to the *E. coli* EPI300 wild-type. Two clones (SUL3 and 5) were resistant to sulphonamide compounds and had reduced susceptibility (but not clinical resistance) to trimethoprim/sulphonamide compared to the EPI300 wild-type. The BACs from these nine clones were sequenced.

The cloned DNA was taxonomically classified by sequence homology and gene synteny: four clones were identified as originating from *Neisseria subflava* (SUL6, SUL8, SUL9, and SUL15), four clones from *Veillonella parvula* (SUL3, SUL5, SUL10, and SUL20), and one clone from *Streptococcus infantis* (SUL11). The size of the inserts ranged from 10,250 bp to 21,161 bp and contained 11 to 20 predicted ORFs, summarised in Table 2.

**Figure 1:**
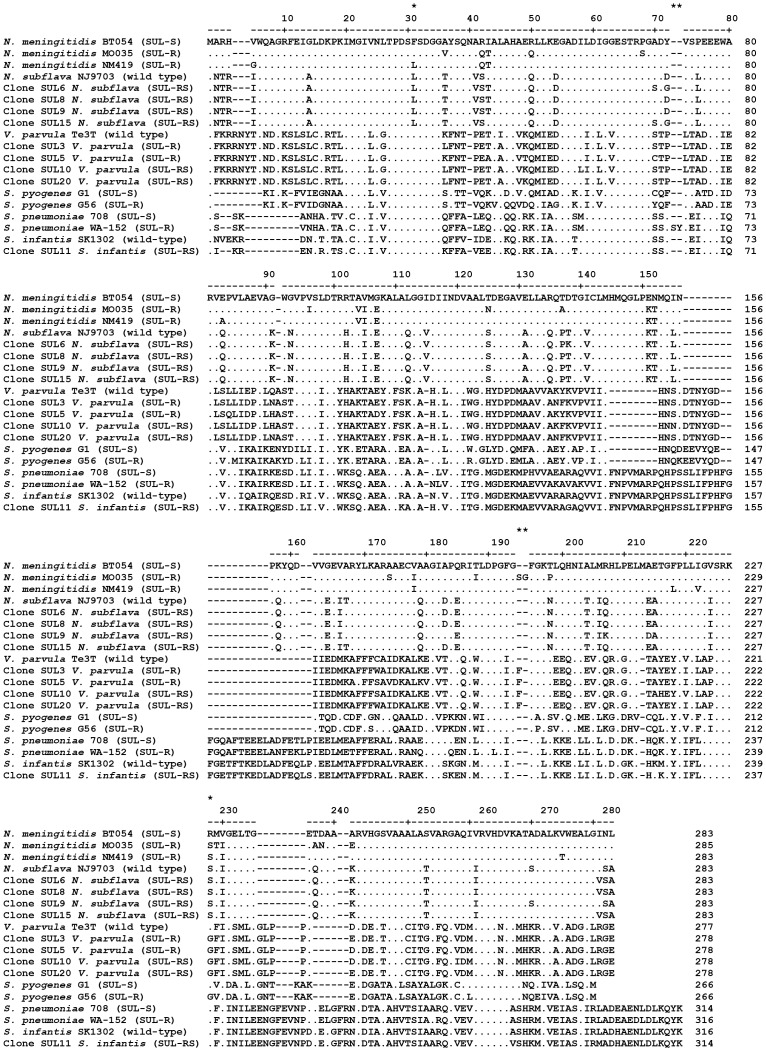
Alignment of the DHPS amino acid sequences from sulphonamide resistant BAC clones and representative DHPS sequences. The numbering above the alignment is based on the DHPS sequence of the *N. meningitidis* strain BT054 and amino acids identical to this sequence are indicated by a dot. Gaps are indicated by a hyphen. Amino acids discussed in the text are indicated by an asterisk above the numbering. SUL-R = sulphonamide resistant; SUL-S = sulphonamide susceptible; SUL-RS = reduced susceptibility to sulphonamide. The nucleotide accession number and reference for the representative DHPS sequences used in the alignments are: *N. meningitidis* BT054 (X68067; [54]), *N. meningitidis* MO035 (X68062; [54]), *N. meningitidis* NM419 (AY722006; [39]), *N. subflava* NJ9703 (ACEO02000001; direct submission), *V. parvula* Te3^T^ (CP001820; [49]), *S. pneumoniae* 708 (U16156; [55]), *S. pneumoniae* WA-152 (AJ311336; [41]), *S. pyogenes* G1 (AJ000686; [40]), *S. pyogenes* G56 (AJ000685; [40]), and *S. infantis* SK1302 (NZ_AEDY01000064; direct submission).

All nine clones possessed the *folP* gene which encodes dihydropteroate synthase (DHPS). DHPS catalyses an essential step in the folic acid biosynthesis pathway and is the target of sulphonamide action [38]. Certain mutant *folP* genes encode a DHPS enzyme that has a lower affinity for sulphonamides, and thus confer reduced susceptibility to this antibiotic. The predicted DHPS amino acid sequences from the BACs were aligned with DHPS sequences of representative *folP* genes (including sulphonamide susceptible and resistant variants) and analysed for the presence of mutations that can confer reduced susceptibility to sulphonamides (Figure 1).

The DHPS of the four *N. subflava* clones had 97.8 to 98.8% amino acid identity to the DHPS encoded by a genome fragment of *N. subflava* (strain NJ9703, accession number ACEO02000001). Two amino acid substitutions were identified in the DHPS sequences of the four clones: a phenylalanine to leucine substitution at amino acid 31 and an arginine to serine substitution at amino acid 228, Figure 1. These mutations have been described previously in *N. meningitidis* and confer resistance to sulphonamides [39]. Both mutations were also present in the *N. subflava* reference DHPS sequence.

For the *V. parvula* clones, the DHPS had 94.4 to 95.6% amino acid identity to the DHPS from the *V. parvula* type strain Prévot Te3^T^ (accession number CP001820). Two mutations with the potential to confer resistance to sulphonamides were identified in these DHPS sequences: insertion of phenylalanine after the glycine at amino acid 189 and an arginine to glycine substitution at amino acid 222. Neither mutation was present in the *V. parvula* type strain. In *V. parvula*, amino acid 189 corresponds to amino acid 194 in *N. meningitidis* (Figure 1), and insertion of two amino acid residues at this position confers resistance to sulphonamides in *N. meningitidis* [39]. Amino acid 222 in the wild-type *V. parvula* DHPS corresponds to amino acid 228 in *N. meningitidis* and mutations at this residue can confer resistance to sulphonamides. Furthermore, at the equivalent position in *Streptococcus pyogenes*, an arginine to glycine substitution confers resistance to sulphonamides [40].

The predicted DHPS amino acid sequence of the single *S. infantis* clone had 93.1% amino acid identity to the DHPS encoded by a genome fragment from *S. infantis* (accession number NZ_AEDY01000064). The DHPS from SUL11 did not possess amino acid substitutions or insertions at the same positions where mutants were identified in the *N. subflava* and *V. parvula* clones (Figure 1). In the related species, *Streptococcus pneumoniae,* amino acid duplications or insertions in the region spanning amino acids 58 to 67 confer resistance to sulphonamides [41], however no such mutations were present in the SUL11 DHPS (Figure 1). Nevertheless a number of amino acid substitutions unique to the SUL11 DHPS were present which have not previously been ascribed to sulphonamide resistant variants of streptococcal DHPS.

The *folP* gene present in each clone is therefore the likely candidate to confer the observed reduced susceptibility or resistance to sulphonamide. The reduced susceptibility or resistance to trimethoprim/sulphonamide of the clones arises because the *E. coli* EPI300 cells are inherently resistant to trimethoprim.

### Composition of the Saliva and Faecal Microbiotas

The microbial profile of each sample was determined by analysis of 16S rRNA gene sequences. From 11,076 to 84,755 sequences were obtained per sample (Table S3), following quality control and removal of OTUs represented by less than five sequences. For the saliva samples, the predominant taxa belonged to Firmicutes (genus *Streptococcus*, *Veillonella*), Proteobacteria (genus *Neisseria*, *Haemophilus*), Bacteroidetes (genus *Prevotella*, *Porphyromonas*), and Fusobacteria (genus Fusobacterium) (Table S3 and Table S4). In the faecal samples the predominant taxa belonged to Bacteroidetes (genus *Bacteroides*, *Prevotella*) and Firmicutes (family *Lachnospiraceae*, *Ruminococcaceae*, genus *Faecalibacterium*, *Roseburia*, *Lachnospira*) (Table S4 and Table S5). The number of unclassified sequences was small in the saliva samples (average 1.9%) but comprised a significant proportion in the faecal samples (average 13.7%) (Table S3 and Table S4). In the saliva DNA used for library construction, the average relative abundances for the genera identified in the activity-based screens were: *Haemophilus* spp. 7.3%, *Neisseria* spp. 9.0%, *Veillonella* spp. 10.8%, and *Streptococcus* spp. 13.9%.

## Discussion

A microarray was employed to rapidly screen the microbiome of each sample for a panel of over 70 well characterised clinically relevant AMR genes. Every sample was positive for one or more AMR genes and in total genes encoding resistance to six antibiotic classes was detected. Many of these genes have a global distribution and have been reported in the human microbiota previously, including *aac6′-lb*, *bla*_TEM_, *bla*_CMY/MOX_, *ereA*, *erm*(B), *strA*, *strB*, *sul2*, *tet*(B), and *tet*(X) [8]–[11], [18], [23], [42]–[44]. These AMR genes generally have broad host ranges and frequently reside on mobile genetic elements such as plasmids and transposons [45]. These properties are likely to have contributed to their wide prevalence and dissemination in human microbiomes. It is also noteworthy that a large number of genes represented on the microarray were not detected in these samples, including, for example, those able to cover plasmid mediated resistance to quinolones and carbapenems.

The microarray enabled a rapid screen for many AMR genes but provided no direct information on their bacterial hosts, genetic context, or whether they are inactivated by point mutations/frameshifts. Additionally, sequenced-based methods such as microarray (and PCR) only allow the detection of known genes. Functional-based screens were therefore undertaken using antibiotics corresponding to those resistance genes identified by microarray. However, in these screens the genes that had been detected by microarray were not recovered. Instead the recovered clones possessed chromosomally located genes, encoding efflux pump proteins or a variant enzyme target of the antibiotic. For clones expressing ampicillin resistance determinants, the *H. parainfluenzae acrRAB* operon encoding a multi-drug efflux pump was recovered. Genes encoding efflux pump proteins have been recovered in other functional-based screens (reviewed in [14]). The cloned predicted transcriptional repressor, AcrR, had <90% amino acid identity to the reference sequence, and may encode an AcrR variant with impaired repressor activity, leading to increased expression of the AcrAB pump. Increased activity of the AcrAB multi-drug efflux pump contributes to the beta-lactamase-negative ampicillin-resistant phenotype observed in some *H. influenzae* clinical isolates [35]. The sulphonamide functional-based screen returned clones from three species, each containing the chromosomally located *folP* gene encoding a mutant DHPS, the target of sulphonamide action. Alterations in the target site of the antibiotic that reduce its binding capacity are a general mechanism for resistance, but, to our knowledge, have not been described previously in clones recovered from functional screens [14].

All the resistance genes recovered by functional-based screening were from commensal but opportunistically pathogenic species from genera which were found by 16S rRNA gene 454 pyrosequencing to represent >7% of the microbiota in the samples studied. Therefore bacteria from these species possess the potential to compromise therapeutic options in the event of disease. Furthermore the genes may be available for acquisition by closely related bacteria, including pathogenic species, via natural transformation, a mechanism of horizontal gene transfer. In *Haemophilus* spp. and *Neisseria* spp., natural transformation is mediated by distinct DNA uptake sequences [46], [47], which were present in multiple copies in each clone from these species. Exchange of DNA between commensal streptococci and the major human pathogen *S. pneumoniae* is also well documented [48] and requires no specific uptake sequences. The *folP* from *V. parvula* encoded a DHPS with novel mutations that gave resistance to sulphonamides, which are not present in the wild-type strain sequence. Resistance to sulphonamides in *V. parvula* has not been extensively investigated [49], although Wüst and Wilkins [50] reported an MIC for co-trimoxazole of four human isolates.

We hypothesise that the recovery of chromosomally located genes in the functional screens reflects the abundance of the sequences present within the microbiomes studied. Although genes such as *sul2* and *bla*_TEM_ were sufficiently abundant to be detected by microarray, they are expected to reside in a diverse set of hosts and genetic environments. Consequently the abundance in any given genetic environment for these genes is low and the use of pooled DNA in the construction of the BAC library would have further diluted this abundance. The microbial profiles obtained in this study were in general agreement with those reported in other studies of the healthy human saliva and faecal microbiomes [42], [51], [52], and showed that the relative abundance of bacterial genera is similar between the different samples so pooling was expected to have had a minimal effect on the relative abundance of chromosomal genes. The use of pooled samples will have also reduced the sensitivity of the microarray assay, allowing the detection of only the most prevalent genes. PCR validated the microarray positive results for four genes; however, some microarray negative samples were PCR positive. This is likely to be a consequence of the greater sensitivity of the PCR method (PCR product accumulation is geometric/exponential, while for microarray labelling product accumulation is arithmetic). In future we would propose using the microarray with DNA preparations from a single subject only.

A powerful advantage of function-based screening is that genes can be recovered without prior knowledge of their sequence. However, a drawback to this approach is that it requires the cloned genes to be expressed and the gene products to be active in the heterologous host, and considerations such as codon usage, promoter sequences and interactions with other proteins can all influence the recovery of clones. For example, the *H. influenzae* AcrAB can confer resistance to several antimicrobials when expressed in *E. coli*, but requires the host encoded TolC protein for this activity [53]. In this study we cloned large fragments of DNA, as this would place any resistance gene identified in context and facilitate identification of the host bacterium. The expression of these cloned genes is therefore likely to be directed by their natural promoters, which must be functional in the *E. coli* host. An alternative strategy is to clone smaller inserts into expression vectors and this can increase clone recovery but provides less information on the origin of the clone.

In this study we have employed two methods to screen for AMR genes in the resistome of healthy humans. The microarray was used as a target-based strategy, to enable a rapid and broad survey of AMR gene content, and provided insight into the diversity of resistances present. However, this approach did not inform on the bacterial hosts possessing these genes, nor on whether the genes detected were intact and expressed in their host. In the functional-based screens intact genes that expressed resistance (or reduced susceptibility) were recovered and the bacterial hosts identified, although this approach has its own limitations (as discussed above). The target- and functional-based approaches we employed have differing shortcomings and advantages; however they can complement each other and together allowed a broad range of resistance genes and mechanisms to be identified. This study provides further evidence that the microbiome of healthy humans harbours a diverse reservoir of resistance mechanisms, some of which are present in populations from several different countries. The techniques described in this study could be employed, in future, to monitor the changes in the resistome in response to antibiotic therapy, and can be employed alongside other methods investigating the microbiota and microbiome.

## Supporting Information

Table S1 **DNA concentrations of samples.**
(XLSX)

Table S2 **Estimation of the sensitivity of the amplification and microarray method using a dilution series of bacterial DNA of known AMR gene content in 500 ng calf thymus DNA.**
(XLSX)

Table S3 **Microarray results obtained with DNA extracts from saliva and faecal samples.**
(XLSX)

Table S4 **Table presenting the number of 16S rDNA gene sequences obtained per sample by high-throughput sequencing and the relative abundance of sequences taxonomically classified to phyla at an even depth of 11070 sequences per sample.**
(XLSX)

Table S5 **Table of the relative abundance of sequences taxonomically classified to genus or next highest possible resolution level (family, order, or class).**
(XLSX)
